# Treating triple negative breast cancer cells with erlotinib plus a select antioxidant overcomes drug resistance by targeting cancer cell heterogeneity

**DOI:** 10.1038/srep44125

**Published:** 2017-03-10

**Authors:** Bin Bao, Cristina Mitrea, Priyanga Wijesinghe, Luca Marchetti, Emily Girsch, Rebecca L. Farr, Julie L Boerner, Ramzi Mohammad, Greg Dyson, Stanley R. Terlecky, Aliccia Bollig-Fischer

**Affiliations:** 1 Barbara Ann Karmanos Cancer Institute, Department of Oncology, Wayne State University, Detroit, MI 48201, USA; 2 The Microsoft Research - University of Trento Centre for Computational and Systems Biology, Rovereto, Italy; 3 Department of Pharmacology, Wayne State University School of Medicine, Detroit MI 48201, USA; 4 Translational Research Institute, Hamad Medical Corporation, Doha, Qatar

## Abstract

Among breast cancer patients, those diagnosed with the triple-negative breast cancer (TNBC) subtype have the worst prog-nosis. TNBC does not express estrogen receptor-alpha, progesterone receptor, or the HER2 oncogene; therefore, TNBC lacks targets for molecularly-guided therapies. The concept that EGFR oncogene inhibitor drugs could be used as targeted treatment against TNBC has been put forth based on estimates that 30–60% of TNBC express high levels of EGFR. However, results from clinical trials testing EGFR inhibitors, alone or in combination with cytotoxic chemotherapy, did not improve patient outcomes. Results herein offer an explanation as to why EGFR inhibitors failed TNBC patients and support how combining a select antioxidant and an EGFR-specific small molecule kinase inhibitor (SMKI) could be an effective, novel therapeutic strategy. Treatment with CAT-SKL—a re-engineered protein form of the antioxidant enzyme catalase—inhibited cancer stem-like cells (CSCs), and treatment with the EGFR-specific SMKI erlotinib inhibited non-CSCs. Thus, combining the antioxidant CAT-SKL with erlotinib targeted both CSCs and bulk cancer cells in cultures of EGFR-expressing TNBC-derived cells. We also report evidence that the mechanism for CAT-SKL inhibition of CSCs may depend on antioxidant-induced downregulation of a short alternative mRNA splicing variant of the methyl-CpG binding domain 2 gene, isoform MBD2c.

Triple negative breast cancer (TNBC) is a molecular subtype that accounts for approximately 15–20% of invasive breast cancer diagnoses in the United States and persons diagnosed with TNBC have the lowest 5-year survival rates among all breast cancer patients. It occurs more prevalently in pre-menopausal women and in African American women^1^,^2^, and obesity is a risk factor for TNBC diagnosis^3^,^4^. TNBC does not express estrogen receptor-alpha, progesterone receptor, or the HER2 oncogene (a member of the epidermal growth factor family of receptor tyrosine kinases); therefore, TNBC lacks targets for effective, molecularly-guided breast cancer therapies. The EGFR oncogene is another member of the epidermal growth factor family, and the concept that EGFR inhibitor drugs could be used as a targeted treatment against TNBC has been put forth based on compelling data estimating that between 30–60% of TNBC express high levels of EGFR^5^,^6^. However, results from clinical trials testing EGFR-targeted inhibitors, alone or in combination with cytotoxic chemotherapy, show little or no improvement in patient outcomes^7^. Thus, it remains that chemotherapy is the only standard of care systemic treatment option for TNBC.

In previous studies we identified that activation of the cell-transforming HER2 oncogene causes induction of intracellular reactive oxygen species (ROS) and activation of redox signaling that impinges on a variety of cancer cell pathways^8^,^9^. We later observed that TNBC cell cultures overexpressing the EGFR oncogene also exhibit aberrantly high levels of ROS. Furthermore, treatment with the antioxidant CAT-SKL in combination with an EGFR-targeted small molecule kinase inhibitor (SMKI) causes a marked growth inhibitory response in TNBC cells that are otherwise resistant to EGFR inhibitors^10^. CAT-SKL is a re-engineered form of the powerful antioxidant enzyme catalase. Previous results indicate that the recombinant enzyme transduces the cell membrane^11^, and this is believed to be mediated by a cell-penetrating peptide sequence^12^. CAT-SKL is distinct from other antioxidant treatments due to its enzymatic reduction of ROS.

In the present study we aimed to ascertain if this novel SMKI plus antioxidant combination treatment strategy may have broad applicability for TNBC and for other breast cancer molecular subtypes. We also aimed to better understand the mechanism for its anti-cancer effectiveness. We studied whether or not CAT-SKL and EGFR SMKI erlotinib were acting on the same cells, or if each agent was targeting a distinct population of cells, i.e., the subset of cancer stem-like cells (CSCs) versus the bulk population of cancer cells. The relevance of CSCs is that they are identified in tumors and breast cancer-derived cell cultures as tumor initiating, self-renewing cancer cells that also give rise to drug resistance and metastatic recurrence^13^. The outcomes of our study suggest that an antioxidant plus EGFR SMKI combined treatment strategy could be specifically developed for treatment of EGFR-expressing TNBC. We report evidence that the EGFR-specific SMKI erlotinib inhibits the non-CSC or bulk TNBC cells and that CAT-SKL inhibits viability of the CSC sub-population. Results of further investigation suggest that CAT-SKL-induced downregulation of the methyl-CpG binding domain 2 gene, the MBC2C isoform specifically, was key to CAT-SKL targeting of CSCs.

## Results

### Effect of combination CAT-SKL plus EGFR-specific or HER2-specific SMKI on breast cancer cell line viability

**Figure 1:**
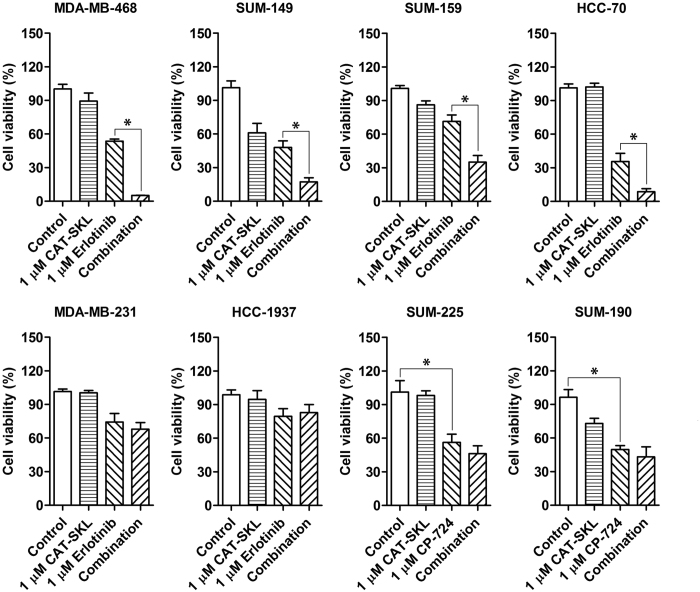
Effect of CAT-SKL and SMKI treatments, alone and in combination, on viability of various breast cancer cell lines. Cell viability was measured following 72 hours of treatment. SUM-225 and SUM-190 are HER2-amplified cell lines that were treated with SMKI CP,724,714 that specifically inhibits HER2. The other cell lines, which express EGFR and represent TNBC, were treated with the EGFR-targeted SMKI erlotinib. Each experiment was run in triplicate and panels are representative of 3 independent experiments, *p < 0.05.

We began our study by testing the effect of the combination treatment, CAT-SKL plus EGFR SMKI or HER2 SMKI, on cell viability across a panel of 8 cell lines. This included six EGFR-expressing, TNBC-derived cell lines and two HER2-amplified breast cancer-derived cell lines. Supplementary Table S1 outlines the molecular characteristics for each of these cell lines, which were previously reported^14^. Results of cell viability assays showed that CAT-SKL or SMKI alone had either a relatively modest effect or no effect, but a significant loss of viability due to three-day combined treatment was observed for four of the six TNBC cell lines, including MDA-MB-468, SUM-149, SUM-159, and HCC-70 (Fig. 1). Each of these four cell lines showed some level of lack of response to erlotinib treatment that was overcome by co-treatment with CAT-SKL. Treatment with CAT-SKL or erlotinib, alone or in combination, appeared to have no effect on TNBC cell lines MDA-MB-231 and HCC1937.

For each of the HER2-amplified breast cancer cell lines tested— SUM-225 and SUM-190— loss of viability was observed for single agent treatment with CAT-SKL, and treatment with HER2-specific SMKI CP724,714 appeared to induce a maximum effect. The response to combination of CP724,714 and CAT-SKL was not significantly different compared to CP724,714 alone (Fig. 1). The results suggest that targeted inhibition of the HER2 oncogene by CP724,714 treatment superseded any potential benefit of CAT-SKL co-treatment.

### CAT-SKL and erlotinib individually act on different sub-populations of TNBC cells

**Figure 2:**
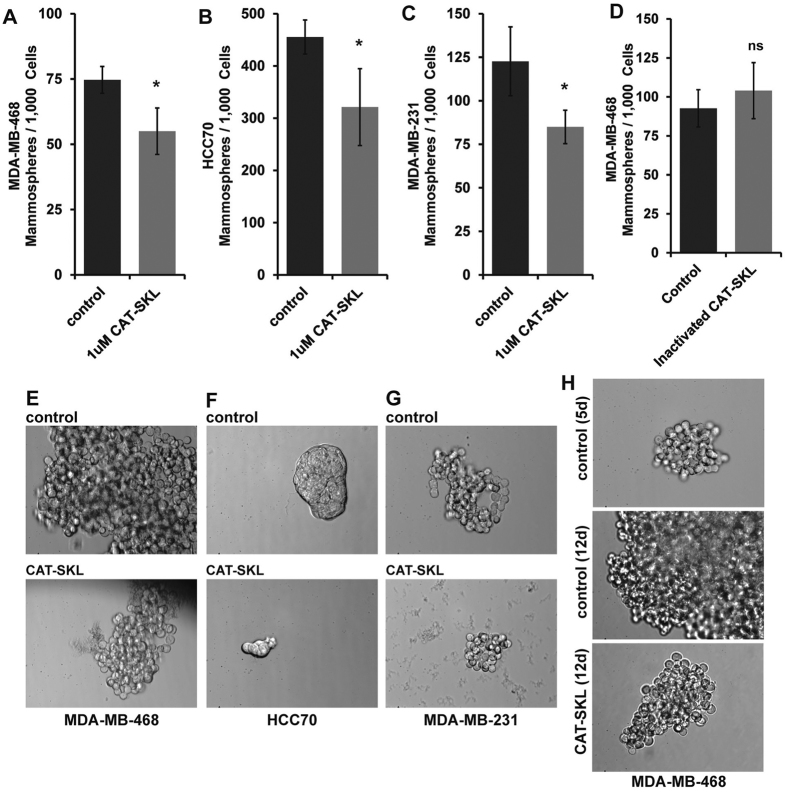
Effect of CAT-SKL on mammosphere formation and self-renewal capacity in CSC-enriched cultures. **(A–C)** A mammosphere assay was used to measure the impact of CAT-SKL treatment on the numbers of mammospheres formed per 1,000 cells passaged, 3 different TNBC cell lines were investigated: MDA-MB-468, HCC70 and MDA-MB-231. **(D)** Confirmation that heat inactivated CAT-SKL does not have the same effect as active CAT-SKL. **(E–G)** Mammosphere images are representative of the effect of CAT-SKL to reduce mammosphere size using MDA-MB-468, HCC70 and MDA-MB-231 TNBC cell lines (40x magnification). **(H)** Effect of CAT-SKL on CSC self-renewal potential using already formed mammospheres, here allowing MDA-MB-468 mammospheres to establish and grow for five days before starting a seven-day course of CAT-SKL treatment. Results showed a marked reduction in mammosphere size (40x magnification). Each experiment was run in triplicate and panels are representative of at least 2 independent experiments, *p < 0.05; ns, not significant.

Next, we investigated if CAT-SKL or erlotinib were targeting distinct sub-populations of cells in TNBC cell line cultures. Using a mammosphere formation assay that calls for culturing breast cancer cells under non-attachment conditions in CSC-selective serum-free media, we tested if CAT-SKL influenced the formation of mammospheres demonstrating the presence of CSCs^15^, in cultures of MDA-MB-468 and HCC70 cell lines. For both of these cell lines, the addition of CAT-SKL to the mammosphere-forming media decreased both the numbers of mammospheres and the size of surviving mammospheres (Fig. 2A,B and E,F). Similar results were observed when we used the same conditions on MDA-MB-231 cells (Fig. 2C,G), which appeared non-responsive to erlotinib and/or CAT-SKL according to viability assay data (Fig. 1). We also tested the effect of CAT-SKL on CSC self-renewal potential using already formed mammospheres, here allowing MDA-MB-468 mammospheres to establish and grow for five days before starting a seven-day course of CAT-SKL treatment. Results showed a marked reduction in mammosphere size (Fig. 2H).

**Figure 3:**
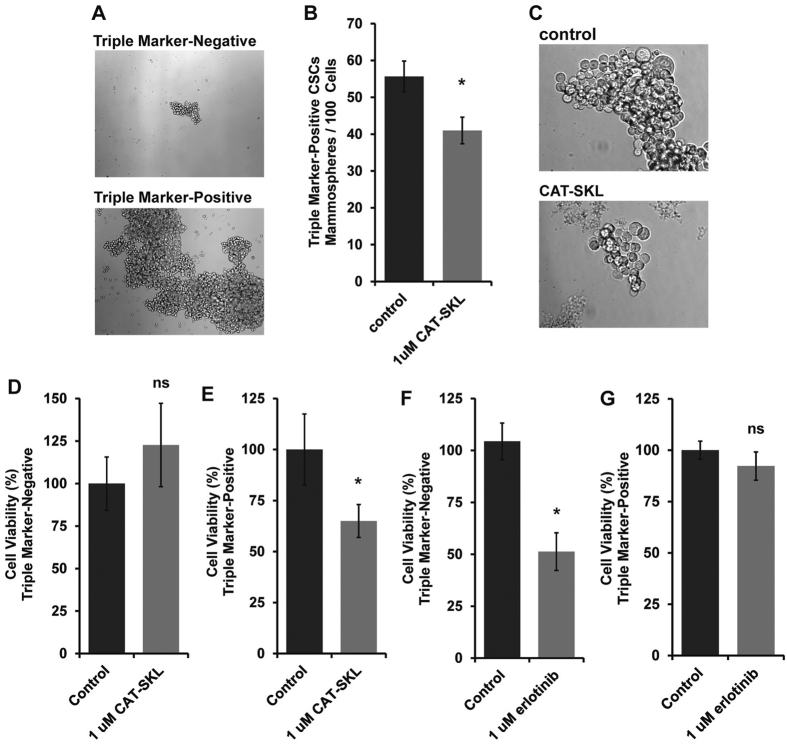
Testing the independent effects of erlotinib and CAT-SKL on CSCs and non-CSC bulk cancer cells isolated from MDA-MB-468 cell cultures. We isolated each cell sub-population from MDA-MB-468 cells by fluorescence-activated cell sorting (FACS). CSCs were segregated based on triple marker-positive status (CD44 + /CD133 + /EpCAM + ); and non-CSC bulk cells were isolated based on absence of all of these markers (i.e., triple marker-negative). (**A**) Triple marker-positive cells thrived in CSC-selective, mammosphere-forming culture conditions and triple marker-negative cells could not be maintained under these conditions (14 days of culture, 10x magnification). (**B**) For triple marker-positive cells maintained in mammosphere-forming conditions, CAT-SKL inhibited mammosphere formation and (**C**) mammosphere growth (40x magnification). In the bottom panels, FACS-isolated sub-populations were immediately re-plated under short-term attached culture conditions to measure the effects of independent CAT-SKL or erlotinib 3 day treatments using the cell viability assay: (**D**) Effect of CAT-SKL treatment on triple marker-negative cells; (**E**) Effect of CAT-SKL on triple marker-positive CSCs; (**F**) Effect of erlotinib treatment on triple marker-negative cells; (**G**) Effect of erlotinib on triple marker-positive CSCs. Each experiment was run in triplicate and panels are representative of at least 2 independent experiments, *p < 0.05; ns, not significant.

To test the independent effects of erlotinib and CAT-SKL on CSCs and non-CSC bulk cancer cells we isolated each cell sub-population by fluorescence-activated cell sorting (FACS). CSCs were isolated based on triple marker-positive status (CD44+/CD133+/EpCAM+); and isolated non-CSC bulk cancer cells expressed none of these cell surface protein markers; i.e., triple marker-negative (CD44/CD133/EpCAM). We confirmed that the triple marker-positive cells thrived in CSC-selective, mammosphere-forming culture conditions and that the triple marker-negative cells could not be maintained under those conditions (Fig. 3A). We also confirmed that CAT-SKL inhibited growth of the triple marker-positive cells maintained in mammosphere-forming conditions (Fig. 3B,C). It was observed in parallel that treatment of triple marker-positive CSCs with CAT-SKL for seven days caused an increase in G2/M checkpoint and subG0 cell cycle arrested fractions and induced cell death (Supplementary Tables S2 and S3).

Finally, we immediately re-plated FACS-isolated sub-populations under short-term attached culture conditions to measure the effects of independent CAT-SKL or erlotinib treatment using the cell viability assay as in Fig. 1. The results showed that CAT-SKL had no effect on triple marker-negative cells, but specifically decreased viability of the triple marker-positive CSCs (Fig. 3D,E). Treatment with erlotinib had the reverse impact; erlotinib specifically and significantly inhibited viability of the bulk triple marker-negative cells and had no effect on CSCs (Fig. 3F,G).

### Discovery of a CAT-SKL-regulated, CSC-supporting factor by analysis of whole genome expression and validation

**Figure 4:**
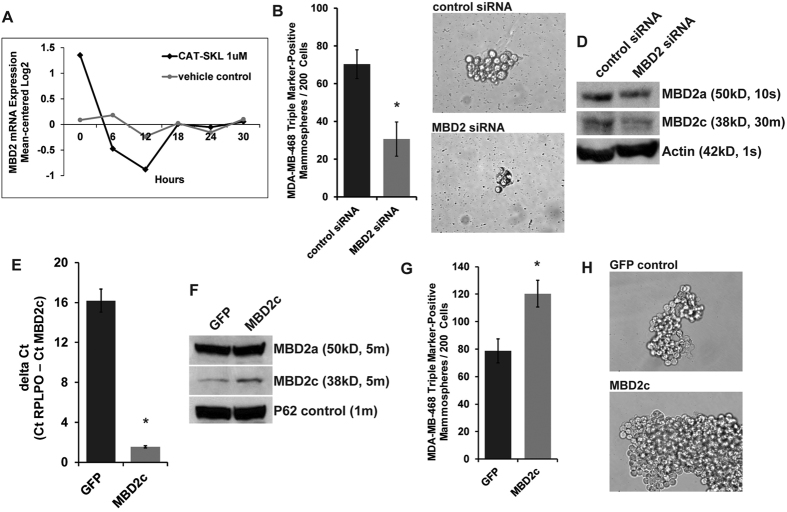
Identification and validation of the potential importance for CAT-SKL regulation of MBD2. (**A**) a time-course microarray approach to measure whole-genome expression identified the methyl-CpG binding domain protein 2 (MBD2) gene as being regulated by CAT-SKL in comparing CAT-SKL-treated and control-treated MDA-MB-468 cell cultures. The time-course experiment collected mRNA data every 6 hours over the course of 30 hours and starting at the zero time-point. **(B–C)** Mammosphere assay culture conditions were used to test the effect of transient siRNA knockdown of MBD2 expression in FACS-isolated, triple marker-positive MDA-MB-468 CSCs (panel C, 40x magnification). **(D)** Confirmation of MBD2 knockdown by immunoblot analysis using beta-actin as a control showed that the siRNA treatment was preferentially downregulating a short form variant. The molecular weight corresponds with alternative mRNA splicing variant known to the NCBI as variant 2 (of 2), also known as MBD2c in the literature. For a measure of MBD2a in whole cell lysates the immunoblot exposure time was 15 seconds (s), for MBD2c the exposure time for the same blot was 30 minutes (m). **(E)** Verification of stable MBD2c overexpression by semiquantitative RT-PCR using RPLPO as the houskeeping control gene (Ct, mean cycle threashold). **(F)** Verification of stable MBD2c overexpression by immunoblot analysis of nuclear lysates using P62 as a control. All immunoblot bands are cropped, full-length blot images are provided in Supplementary Figure S7. **(G,H)** Mammosphere assay culture conditions were used to test the effect of stable MBD2c overexpression in MDA-MB-468 cells relative to green fluorescent protein (GFP)-expressing MDA-MB-468 control cells (panel H, 40x magnification). The siRNA knockdown and stable overexpression experiments were done twice, independently and in triplicate, *p < 0.05; ns, not significant.

For insights as to what gene(s) CAT-SKL may be regulating to impact the CSC phenotype we used a microarray approach to measure whole-genome expression in CAT-SKL-treated and control-treated MDA-MB-468 cell cultures. We conducted a time-course experiment collecting mRNA data every 6 hours over the course of 30 hours of treatment and starting at the pre-treatment, zero time-point. The objective of our data analysis approach described in Methods was to identify gene transcripts showing time-dependent expression level changes (greater than two-fold, log2) only in the CAT-SKL-treated condition. These criteria identified 21 candidate genes (Supplementary Figure S4). Based on compelling data in the literature to support that the CSC phenotype is modulated at the level of gene transcription, reviewed in ref. 16, we assessed our resulting gene set to identify transcriptional regulators and selected the methyl-CpG binding domain 2 (MBD2) gene for further investigation. The data for MBD2 displayed a significant and sustained decreased expression in the CAT-SKL-treated condition and showed no change in the control cultures (Fig. 4A).

Hypothesizing that downregulation of MBD2 gene expression was a key factor in CAT-SKL inhibition of CSC viability and self-renewal, we used mammosphere assay culture conditions to test the effect of transient siRNA knockdown of MBD2 expression in FACS-isolated, triple marker-positive MDA-MB-468 CSCs. We used two commercially available pre-validated MBD2-targeted siRNA constructs independently, and for each the result was a significant reduction in the numbers of viable mammospheres and reduced mammosphere size (Fig. 4B,C and Supplementary Figure S5). In confirming MBD2 knockdown by immunoblot analysis we observed that the siRNA treatment was preferentially downregulating a short form variant (Fig. 4D and Supplementary Figure S5). Its immunoblot estimated molecular weight corresponds with an alternative mRNA splicing variant known to the NCBI as variant 2 (of 2), also known as MBD2c in the literature^17^. These data suggested that the CSC-inhibiting effect of the MBD2-targeted siRNA treatment was linked to downregulation of the short isoform MBD2c. To further test this and the role of MBD2c specifically in promoting CSCs, we proceeded to stably overexpress the MBD2c variant in MDA-MB-468 cells (Fig. 4E,F), and observed that this caused a significant increase in the numbers of viable mammospheres and an increase in mammosphere size (Fig. 4G,H).

**Figure 5:**
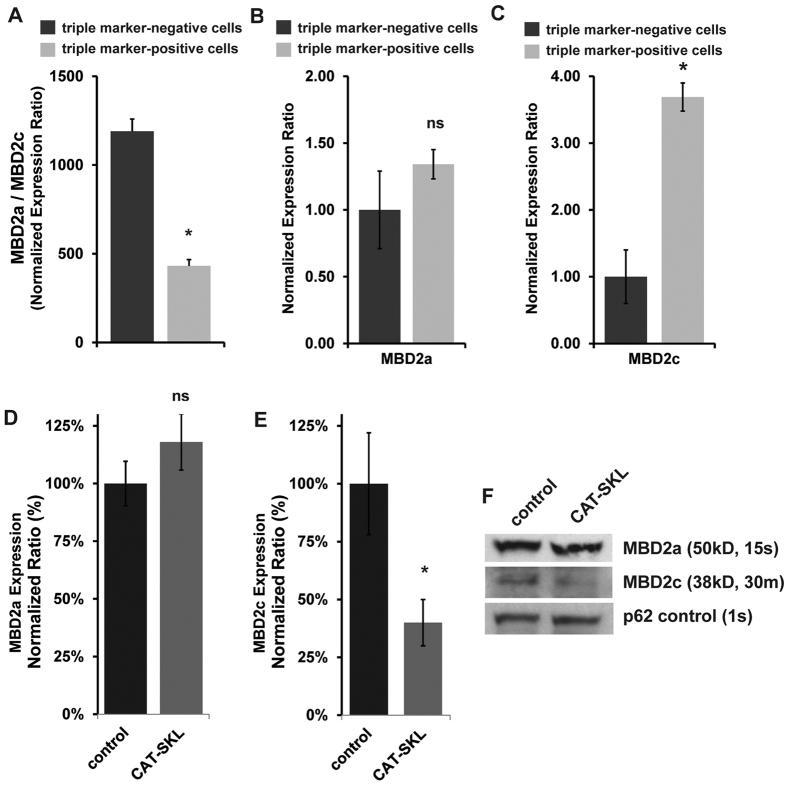
Measure of MBD2 isoform levels in triple marker-negative non-CSCs and in triple marker-positive CSCs and the effect of CAT-SKL on isoform levels. Using the MDA-MB-468 cell line, the relative mRNA levels of MBD2a and MBD2c were measured in FACS-isolated triple marker-negative bulk cells and in triple marker-positive CSCs. Semiquantitative RT-PCR analysis showed that (**A**) MBD2a transcript levels were by far greater than MBD2c in both cell sub-types, and (**B**) MBD2a was expressed at nearly the same levels in both cell niches. (**C**) In contrast, a comparison of MBD2c transcript levels showed that MBD2c was nearly four times higher in triple marker-positive CSCs relative to triple marker-negative cells. (**D**) The effect of CAT-SKL on MBD2a and (**E**) MBD2c isoform expression in MDA-MB-468 FACS-isolated triple marker-positive CSCs according to semi-quantitative RT-PCR, and by (**F**) immunoblot methods using nuclear protein isolate. For MBD2a the immunoblot exposure time was 15 seconds (s), for MBD2c the exposure time for the same blot was 30 minutes (m). Each bar graphed experiment was run in triplicate and panels are representative of at least 2 independent experiments, *p < 0.05; ns, not significant. The immunoblot experiment was independently done twice.

Characterization of MBD2 isoform levels in FACS-sorted triple marker-negative non-CSCs and in triple marker-positive CSCs, and whether or not CAT-SKL preferentially impacted either one of the isoforms became the next important issues to address. Using the MDA-MB-468 cell line, we measured the relative mRNA levels of MBD2a and MBD2c in FACS-isolated triple marker-negative bulk cells and in triple marker-positive CSCs. Here we learned that MBD2a transcript levels were always greater than MBD2c (Fig. 5A), and that MBD2a was expressed at nearly the same levels in both cell niches (Fig. 5B). In contrast, a comparison of MBD2c transcript levels showed that MBD2c was nearly four times higher in triple marker-positive CSCs relative to triple marker-negative cells (Fig. 5C). Thus, while MBD2a levels were at a similar level in both cancer cell subtypes, MBD2c was significantly higher in the triple marker-positive CSC fraction.

We moved forward to measure the effect of CAT-SKL on MBD2 isoform expression in FACS-isolated triple marker-positive CSCs. According to both RT-PCR and immunoblot methods CAT-SKL treatment significantly reduced MBD2c levels, but had no detectable impact on high-level MBD2a expression (Fig. 5D–F).

### Regulation of triple marker-positive CSCs and the MBD2c isoform is recapitulated with application of antioxidant (−)-epicatechin

**Figure 6:**
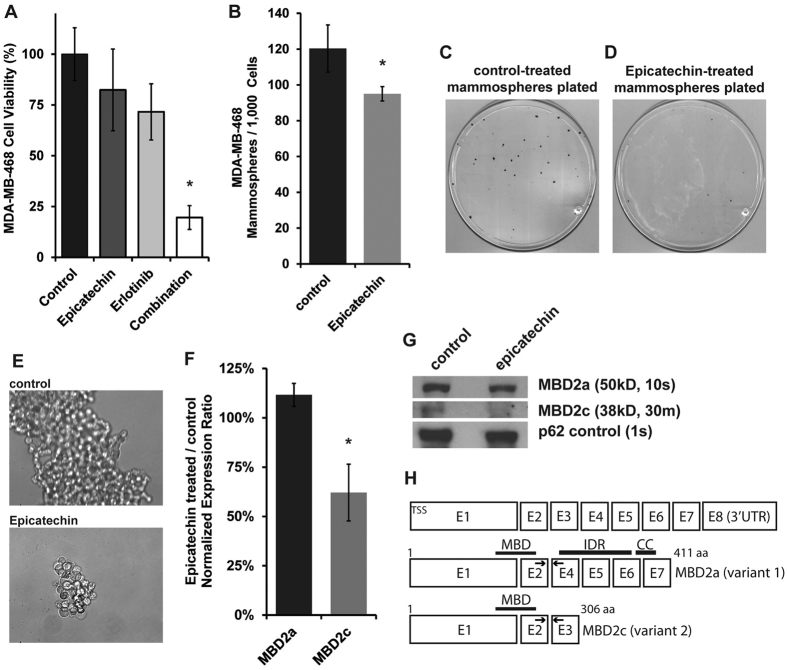
Regulation of triple marker-positive CSCs and the MBD2c isoform by (−)-epicatechin. (**A**) (−)-epicatechin (100 uM) alone had no significant effect on viability of MDA-MB-468 cell cultures in attached growth conditions. The combination of (−)-epicatechin plus erlotinib, however, did significantly decrease cell viability. **(B–E)** Alone, (−)-epicatechin also potently decreased mammosphere-formation and growth (panel E, 40x magnification). **(F,G)** Using FACS-isolated triple marker-positive MDA-MB-468 cells demonstrated that (−)-epicatechin treatment caused a reduction of MBD2c mRNA and protein levels, and that MBD2a levels were unchanged. For a measure of MBD2a in nuclear protein isolates the immunoblot exposure time was 15 seconds (s), for MBD2c the exposure time for the same blot was 30 minutes (m). **(H)** Representation of the MBD2 gene and coding structures and exons (**E**) for alternative mRNA splicing variants MBD2a and MBD2c (NCBI variants 1 and 2, of 2); arrows represent the location of primers used in semi-quantitative RT-PCR analysis. Each bar graphed and mammosphere experiment was run in triplicate and panels are representative of at least 2 independent experiments, *p < 0.05; ns, not significant. The immunoblot experiment was independently done twice.

The final sets of data presented are from experiments designed to learn if treatment with other antioxidants could replicate the effects of CAT-SKL (1 uM) to inhibit growth of self-renewing CSCs and downregulate MBD2c in TNBC cell cultures. We tested a range of subnanomolar to micromolar concentrations of resveratrol and N-acetyl-L-cysteine (NAC) on MDA-MB-468 cells, and none replicated the effects of CAT-SKL (data not shown). Using (−)-epicatechin (100 uM) we observed that alone it had no significant effect on viability of MDA-MB-468 cell cultures in attached growth conditions. The combination of (−)-epicatechin plus erlotinib, however, did significantly decrease cell viability (Fig. 6A). Alone, (−)-epicatechin also potently decreased mammosphere-formation and growth (Fig. 6B–E). Finally, (−)-epicatechin treatment of MDA-MB-468 cell cultures caused a reduction of MBD2c mRNA and MBD2c protein levels, while MBD2a levels were unchanged (Fig. 6F,G).

## Discussion

In the coming years, new advances in precision cancer medicine will depend on combining drugs to target the heterogeneity of tumor cells to improve patient survival outcomes. Moreover, to overcome the persistence of de novo and developed drug resistance, more effective therapeutic strategies must target the most aggressive subset of cancer cells that give rise to metastatic recurrence^16^. These clinical challenges underscore the value of the present study. We found that treatment with the antioxidant CAT-SKL selectively impacted CSCs and treatment with the SMKI erlotinib targeting EGFR selectively impacted non-CSCs. Thus, combining the antioxidant CAT-SKL with the SMKI erlotinib targets both CSCs and bulk cancer cells in cultures of EGFR-expressing TNBC-derived cell lines.

The results from testing the SMKI plus CAT-SKL antioxidant combination on a set of molecularly diverse breast cancer-derived cell lines suggest that the treatment strategy could be of substantive benefit to patients if developed as a molecularly targeted therapy for TNBC that overexpresses EGFR. However, the data also indicate that CAT-SKL will not provide added anti-cancer benefit when another oncogene, such as HER2, is the molecular co-target. This interpretation of our HER2-amplified cell line data is in line with understanding that the HER2 oncogene has a role in promoting CSCs and that inhibition of HER2 signaling reduces CSCs in tumors^18^.

We report evidence that the mechanism for CAT-SKL inhibition of CSCs in TNBC cultures may depend on antioxidant-induced downregulation of epigenetic reader MBD2, more specifically isoform MBD2c. A recent report by Lu *et al*. delineates opposing functions for MBD2a and MBD2c in human pluripotent stem cells (hPSCs)^17^. Their data support the mechanistic model where in hPSCs MBD2a binds methyl-CpG promoter sequence and recruits the Nucleosome Remodeling and Deacetylase co-repressor complex (NuRD) to silence transcription of genes required for pluripotency, thus MBD2a promotes hPSC differentiation. Furthermore, in hPSCs it appears that MBD2c binds the same densely methylated promoter sequences^17^; and because MBD2c lacks the C-terminal coiled-coil domain required to recruit the NuRD complex as depicted in Fig. 6H 19, upregulated MBD2c competes with MBD2a for methyl-CpG DNA binding to activate genes and promote pluripotency in hPSCs^17^. As such, our findings that implicate MBD2c in the promotion of TNBC CSCs are novel, yet provide another example of how signaling pathways in CSCs and hPSCs may be similar^20^.

On the other hand, our data also indicate that there is likely to be a distinction for MBD2c function in CSCs in comparison to hPSCs. In studying TNBC CSCs we observe that levels of MBD2a are far higher than MBD2c levels, approximately four- to five-hundred times higher according to semiquantitative RT-PCR analysis and our immunoblot data are in-line with that (noting that to achieve similar band intensity, using the same blot and an antibody that detects both^17^, the exposure time for MBD2a was less than thirty seconds while the MBD2c exposure time was thirty minutes). This suggests that a co-occupying, competitive methyl-CpG DNA binding model may not extend to TNBC CSCs. Moreover, compared to MBD2a, MBD2c lacks both the NuRD-recruiting coiled-coil domain and the intrinsically disordered region (IDR). Presence of the IDR increases the binding affinity of MBD2 for methylated DNA^21^. Altogether, these findings raise the idea that MBD2c may regulate a transcriptional program independent of MBD2a in TNBC CSCs. This is a line of investigation we intend to pursue. Further analysis will also be required to understand how treatment with CAT-SKL causes a reduction in MBD2c transcript and protein, possibly by mechanisms that undermine transcript stability or that impinge on the process of alternative MBD2 mRNA splicing.

Our work also tested multiple antioxidants and identified a second that recapitulated the effects of CAT-SKL on TNBC CSCs. More work needs to be done to understand why, but only select antioxidants appear to be effective. CAT-SKL at 1 uM delivers catalase enzyme function that targets hydrogen peroxide and (−)-epicatechin used at 100 uM is a flavonoid that is known to chemically react with hydrogen peroxide^22^, and both downregulated MBD2c in CSCs and inhibited self-renewing CSC growth. They each also were shown to reduce intracellular and extracellular hydrogen peroxide levels (Supplementary Figure S6). This report underscores the need for future research to learn more about the role of ROS signaling in tumor initiating cancer cells. Moreover, further research is needed to understand how CAT-SKL, (−)-epicatechin and reduction of hydrogen peroxide function— regarding both the site of action and impact on signaling pathways— to affect MBD2c and CSCs. Nonetheless, as both (−)-epicatechin and CAT-SKL have been used therapeutically in mice^11^,^23^,^24^, pre-clinical research into the potential utility of combining an antioxidant with an anti-EGFR drug in treating breast cancer may be relatively straight forward.

We and others have shown that increased intracellular ROS levels in breast cancer cells have a signaling role that promotes breast carcinogenesis and cancer progression^9^,^25^. Also, increased levels of ROS in cancer cells are tightly regulated— kept in check by upregulation of enzymes such as superoxide dismutase (SOD)— in order for cancer cells to survive^26^. It remains to be seen how and under what molecular circumstances oncogenic ROS can be effectively targeted to treat breast cancer. Research has been ongoing from the perspective that inhibition of SOD, which converts the more reactive superoxide radical 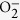 to less reactive hydrogen peroxide, can cause the accumulation of superoxide radicals causing cell damage leading to cancer cell death^26^. Our investigation into a reengineered catalase biologic offers new molecular insight for breast cancer. In summary, the results indicate that a decrease in hydrogen peroxide by CAT-SKL treatment in combination with an EGFR inhibitor holds promise of a novel, therapeutic modality to treat TNBC, a breast cancer subtype with the worst prognosis and high need for treatment options.

## Methods

### Cell lines and culture conditions and treatment agents

The previously established human triple negative breast cancer and HER2-positive breast cancer cell lines were recently acquired for this study from the American Type Culture Collection (ATCC, Manassas, VA) or from the Biobanking and Correlative Sciences Core (BCSC) at the Karmanos Cancer Institute (KCI, Detroit, MI). The BCSC provides a record of authenticity for each cell line lot based on short tandem repeat (STR) analysis. HCC-70 cells were maintained in 5% FBS RPMI-1640 media containing amphotercin B and gentamycin at 37 °C, 5% CO_2_. All other cells were maintained in 5% FBS DMEM media containing amphotercin B and gentamycin at 37 °C, 5% CO_2_. Erlotinib, CP724,714 and -(−)epicatechin were from Sigma-Aldrich (St. Louis, MO). We previously showed that CP724,714, which was originally developed by Pfizer Inc. (Groton, CT), specifically inhibits HER2 activity^9^. CAT-SKL is a reengineered form of the antioxidant enzyme catalase, formulated and prepared as described previously^11^,^12^,^27^,^28^,^29^.

### Measurement of Hydrogen Peroxide

The activity of CAT-SKL to reduce hydrogen peroxide in breast cancer cell cultures was confirmed using two distinct assays. The first known as the Amplex Red Assay kit (Thermo Fischer Scientific, Waltham, MA) measured extra-cellular hydrogen peroxide. Performed following the manufacturers instructions, 2000 cells were seeded in 100 uL of 5% FBS DMEM media onto wells of a 96-well plate and cultured for 18 hours. Then, media was replaced with the same media or the same media with 1 uM CAT-SKL. Hydrogen peroxide levels were measured after 24 hours of treatment using a Synergy 2 plate reader (Biotek, Shoreline, WA). The second approach measured intracellular hydrogen peroxide levels using the MAK164 Intracellular Assay kit (Sigma-Aldrich). Here following the manufacturers protocol, after 24 hours CATSKL-containing media was washed away by a PBS rinse before the hydrogen peroxide sensor solution was applied and measurements using a Synergy 2 plate reader were taken after a 4 hour incubation. Supplementary Figure S6 displays the significant impact of CAT-SKL and -(−)epicatechin treatments on hydrogen peroxide levels in breast cancer cell cultures.

### Cell viability assay (attached cultures)

Cell viability was measured using the CellTiter-Glo luminescent assay kit (Promega, Madison, WI), following the manufacturers instructions. 3,000 cells were seeded per well in 100 uL of culture media in 96-well plates. The next day, the media was changed and the compounds were added by dilution in the fresh media. Viability was measured after 3 days of treatment using a Synergy 2 plate reader.

### Mammosphere formation assay

The presence of and self-renewal capacity of cancer stem cells (CSCs) was examined in breast cancer triple negative cell lines by the mammosphere formation assay, as described previously^15^. Briefly, 1,000 single cells were seeded in 1.5 mL of the FBS-free sphere formation media (1:1 DMEM:F-12 media plus with B-27 and N-2 supplements, Gibco/Life Technologies) in 6-well Ultra Low Attachment plates (Corning Inc., Corning, NY). The treatments were replenished every 3 days. After 7 days of incubation, the mammospheres (equal or greater than 50 micrometer diameter) were counted and reported as a fraction of the total number of cells seeded. To assess self-renewal capacity mammospheres were allowed to form and grow for 5 days. Then, treatments were added and replenished every 3 days after. After 7 days of treatment the mammospheres were counted. Images were taken using a Nikon Eclipse TE2000-U microscope.

### Isolation of CSC triple-marker positive cells

To test the independent effects of erlotinib and CAT-SKL on CSCs and non-CSC bulk cancer cells we isolated each cell sub-population by fluorescence-activated cell sorting (FACS), which was done at the Karmanos Cancer Institute Microscopy, Imaging and Cytometry Resources Core (KCI MICR). CSCs were segregated based on triple marker (CD44+/CD133+/EpCAM+) positive status; and isolated non-CSCs expressed none of these markers (i.e., triple marker-negative). We confirmed that the triple marker-positive cells thrived in CSC-selective, mammosphere-forming culture conditions and that the triple marker-negative cells could not be maintained under those conditions. Isolated triple marker-positive CSCs were cultured in the FBS-free mammosphere formation media to maintain the undifferentiated status. Fluorochrome-labeled monoclonal antibodies against human CD44, CD133, and EpCAM proteins were obtained from EBiosciences (San Diego, CA), Miltenyl Biotec (Cologne, Germany), and BD Biosciences (Franklin Lakes, NJ), respectively.

### Cell cycle analysis

DAPI-staining and flow cytometric assay was conducted at the KCI MICR Core to assess the cell cycle under different experimental conditions. After washing, the cells were fixed with 4 mL of 75% ethanol at −20 °C overnight. The fixed cells were washed and stained with DAPI solution (Sigma, 1 mg/mL stock solution) at 4 °C for 3 h, followed by flow cytometric analysis.

### Apoptosis assay

Annexin V-PI-staining/flow cytometric assay was conducted to examine the effect of CAT-SKL on apoptosis in CSC-like cells. Briefly, after treatment, the cells were harvested, washed with PBS solution once, and further processed to apoptosis assay by using FITC Annexin V/Dead Cell Apoptosis kit (Thermo Fisher Scientific, Waltham, MA), and FACS analysis at the KCI MICR Core.

### Semi-Quantitative RT-PCR of mRNA

We conducted semi-quantitative RT-PCR analysis as previously^9^. RNA was extracted from cells using the Qiagen (Valencia, CA) RNeasy kit. RNA was converted into cDNA via a reverse transcription reaction using oligo dt primers and the qScript cDNA Synthesis kit (Quanta Biosciences, Beverly, MA). 20 uL reactions were run in 96-well plates, using 100ng cDNA and SYBR Green Master Mix (Thermo Fischer Scientific). Reactions were run in triplicate using the StepOnePlus Real-Time PCR System (Thermo Fischer Scientific). Primer pairs specific to MDB2a or MDB2c were previously reported in ref. 17. Actin was used as the reference gene. All primers were from Integrated DNA Technologies Inc. (Coralville, IA). Relative quantification was calculated using the delta-delta Ct method^30^.

### Statistical analysis

The cell line data are presented as the mean and standard deviation of a representative experiment. Semiquantitative RT-PCR data are presented as the mean of multiple experiments with the standard error of the mean. Student T-test was performed to test the significance of difference between two groups, a p-value equal or less than 0.05 is considered to be statistically significant.

### Time-course, whole genome expression profiling by microarray analysis

Whole genome expression analysis was conducted as we have done previously^9^,^31^. Briefly, MDA-MB-468 cell cultures were treated with CAT-SKL (1 uM) or vehicle control (media diluent), and RNA was isolated using the RNeasy Kit (Qiagen, San Diego, CA) from parallel plated cultures at 0 h and every 6 hours after start of treatment for 30 hours, yielding 6 time points. Expression levels were determined by microarray analyses using the Illumina human HT-12v4 array (Illumina, San Diego, CA). Data were processed for quality control in GenomeStudio (detection p-value <0.01), background subtracted, log2 transformed and quantile normalized. Raw and analyzed data sets are available through NCBI Gene Expression Omnibus accession number GSE69799.

We applied a filtering method to the log2, normalized expression data, introduced by us previously^31^, which separates time-course gene expression profiles that can be fit to linear models from chaotic expression profiles. We further filtered this result to identify 21 genes showing fold-change ≥2 log2 in the course of CAT-SKL treatment, and fold-change ≤1.5 log2 in the control time-course.

### Transfection of MBD2 and control siRNA in breast cancer cells

A pre-validated human MBD2 siRNA constructs (catalogue ID numbers s17079 and s17080) and a control non-silencing, scrambled construct (catalogue number 4390846) were purchased from Thermo Fisher Scientific (Waltham, MA). MDA-MB-468 cells were transfected with these constructs using DharmaFECT reagent also from Thermo Fisher Scientific, following the manufacturer protocol.

### Stable overexpression of MBD2c in breast cancer cells

Packaged lentiviral particles to overexpress GFP and MBD2c were purchased from Cyagen Biosciences (Santa Clara, CA). The human MBD2c (NM015832.4) gene was custom synthesized and subcloned into a lentiviral expression vector downstream of a CMV promoter region (same as for GFP). The construct was sequenced to ensure that the sequence and orientation were correct. Similar to what we have done before^8^, MDA-MB-468 cells were transduced with lentivirus and selected with puromycin. MBD2c expression levels were detected using semi-quantitative RT-PCR and Western blotting.

### Immunoblot analysis

Immunoblot analysis to detect the relative levels of MBD2 proteins in whole cell lysate or nuclear extracts was performed as we have previously done^9^. Briefly, 50 ug of total protein from each sample were loaded and separated on 10% SDS-PAGE gels, and then transferred to a nylon membrane using Mini Trans-Blot Electrophoretic Transfer Cell (Bio-Rad, Hercules, CA). Membranes were probed with primary antibodies following suppliers recommendation and secondary peroxidase-conjugated antibodies (anti-mouse or rabbit). Primary antibodies included: polyclonal anti-human MBD2 (Bethyl Laboratories, Montgomery, TX) that was used previously^17^, and monoclonal beta-actin and monoclonal nucleoporin P62 (both Sigma-Aldrich) for whole cell and nuclear extract controls, respectively.

## Additional Information

**How to cite this article:** Bao, B. *et al*. Treating triple negative breast cancer cells with erlotinib plus a select antioxidant overcomes drug resistance by targeting cancer cell heterogeneity. *Sci. Rep.* **7**, 44125; doi: 10.1038/srep44125 (2017).

**Publisher's note:** Springer Nature remains neutral with regard to jurisdictional claims in published maps and institutional affiliations.

## Supplementary Material

Supplementary Information
