# Supplementary material for: Treating triple negative breast cancer cells with erlotinib plus a select antioxidant overcomes drug resistance by targeting cancer cell heterogeneity
Source: Sci Rep. 2017 Mar 10;7:44125. doi: 10.1038/srep44125 (PMC5345072; doi:10.1038/srep44125)
Supplement: Supplementary Information [file srep44125-s1.doc]

**Treating triple negative breast cancer cells with erlotinib**

**plus a directed antioxidant overcomes drug resistance by targeting cancer cell heterogeneity**

**Bin Bao, Cristina Mitrea, Priyanga Wijesinghe, Luca Marchetti, Emily Girsch,**

**Rebecca L. Farr, Julie L Boerner, Ramzi Mohammad, Greg Dyson, Stanley R.**

**Terlecky, and Aliccia Bollig-Fischer**

| **Supplementary Table S1.** Summary of molecular subtype, originating tissue histology and oncogene and tumor suppressor aberration data for breast cancer cell lines tested in culture with erlotinib or CP724,714 plus CAT-SKL combination treatment strategy. | | | |
| --- | --- | --- | --- |
| **Cell Line Name** | **Subtype** | **EGFR**  **Amp and/or OE** | **Other Gene Mutations/CNV** |
| MDA-MB-468 | TNBC-BL1 | YES | PTEN, RB1, SMAD4, TP53 |
| SUM149 | TNBC-BL2 | YES | BRCA1, TP53 |
| SUM159 | TNBC-MSL | YES | HRAS, PIK3CA, TP53 |
| HCC70 | TNBC-BL2 | YES | PTEN, TP53 |
| MDA-MB-231 | TNBC-MSL | YES | BRAF, KRAS, PDGFRA, CDKN2A, NF2, TP53 |
| HCC1937 | TNBC-BL1 | YES | BRCA1, MAPK13, MDC1, TP53 |
| SUM225 | HER2 AMP-BL1 | no | TP53 |
| SUM190 | HER2 AMP-Luminal | no | PIK3CA, FGFR2, TP53 |
| **Abbreviations:** ANC, anaplastic carcinoma; Amp, gene copy number amplification; BC, breast cancer; BL1, basal-like 1; BL2, basal-like 2; CNV, copy number variation; DC, ductal carcinoma; IDC, invasive ductal carcinoma; INF, inflammatory breast cancer; MSL, mesenchymal like; met, metastatic lesion; OE, overexpression; TNBC, triple negative breast cancer.  **Citation:** Kao J, et al. Molecular profiling of breast cancer cell lines defines relevant tumor models and provides a resource for cancer gene discovery. *PLoS One* 4: e6146, 2009. | | | |

**Supplementary Table S2.** Effect of CAT-SKL treatment on apoptosis in FACS-sorted MDA-MB-468 triple marker-positive cells

|  | **Control**  **(mean ± SD)** | **CAT-SKL treated (mean ± SD)** | **P values (n=3)** |
| --- | --- | --- | --- |
| **Alive (%)** | 59.13±1.37 | 46.80±1.15 | 0.0003 |
| **Early apoptotic (%)** | 2.22±0.25 | 3.53±0.12 | 0.0011 |
| **Late apoptotic (%)** | 17.27±1.07 | 25.17±0.76 | 0.0005 |
| **Necrotic (%)** | 17.4±0.75 | 20.03±0.64 | 0.0099 |

Ten thousand triple marker-positive (CD44+/CD133+/EpCAM+) MDA-MB-468 CSCs were seeded in sphere formation media in each well of 6-well ultra-low attachment plates (Corning). The media included CAT-SKL (1 uM). After 7 days, the cells were harvested to assay for cell death using Annexin V/PI staining and fluorescence activated flow cytometry in order to sort and count cell fractions.

**Supplementary Table S3.** Effect of CAT-SKL treatment on cell cycle progression in FACS-sorted MDA-MB-468 triple marker-positive cells

|  | **Control**  **(Mean ± SD)** | **CAT-SKL**  **(Mean ± SD)** | **P values (n=3)** |
| --- | --- | --- | --- |
| **G0-G1 (%)** | 49.87±0.62 | 44.54±0.35 | 0.0002 |
| **G2/M (%)** | 29.58±0.60 | 33.72±0.95 | 0.0031 |
| **S phase (%)** | 20.56±0.13 | 21.73±1.13 | 0.1477 |

Ten thousand triple marker-positive (CD44+/CD133+/EpCAM+) MDA-MB-468 CSCs were seeded in sphere formation media in each well of 6-well ultra-low attachment plates (Corning). The media included CAT-SKL (1 uM). After 7 days, the cells were harvested for cell cycle assay with DAPI staining flow cytometry.


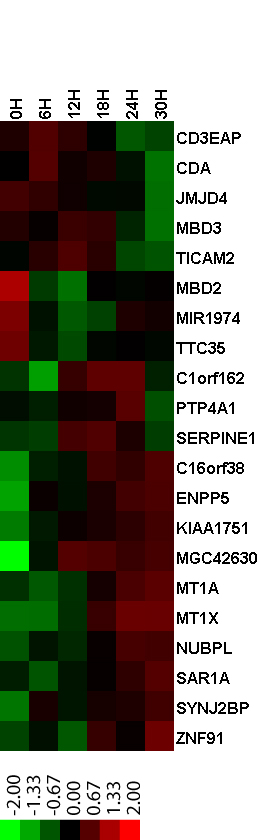


**Supplementary Figure S4**. Resulting gene set and expression levels (mean-centered, log2) from analysis of time-course (6 time points, 0 hour-30 hour), whole-genome microarray analysis of CAT-SKL-treated MDA-MB-468 cell line cultures. The 21 identified genes showed time-dependent mRNA expression level changes (greater than two-fold, log2) only in the CAT-SKL-treated condition.

**Supplementary Figure S5.** Transient knockdown of MBD2 in FACS isolated MDA-MB-468 triple marker positive CSCs using a second, independent MBD2-targeted siRNA construct. **(A)** Immunoblot analysis again showed that siRNA treatment was preferentially downregulating the short form variant, MBD2c. **(B-C)** The MBD2-targeted siRNA treatment had a significant inhibitory effect on mammosphere formation (p<0.05), and growth (40x magnification).


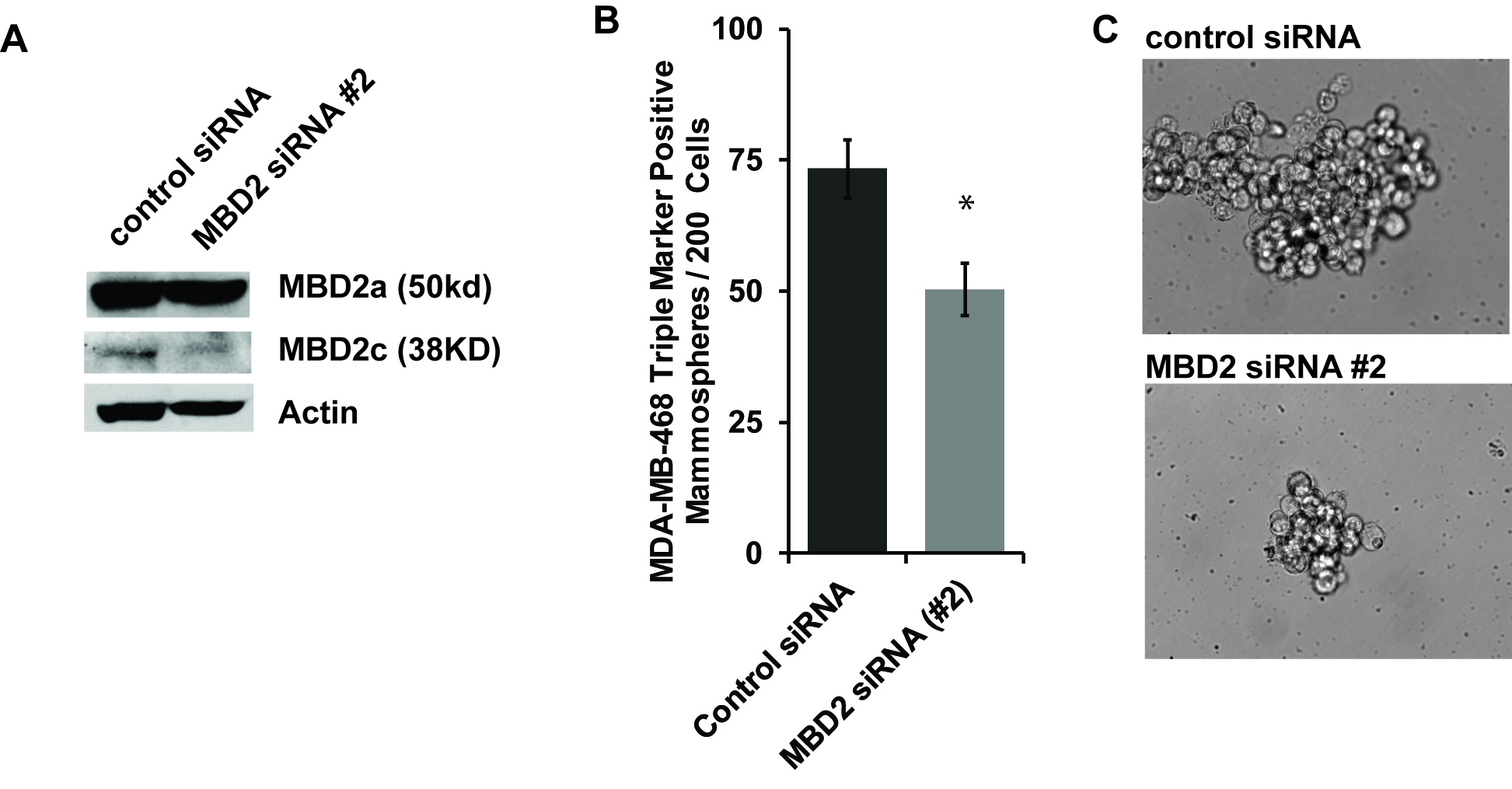

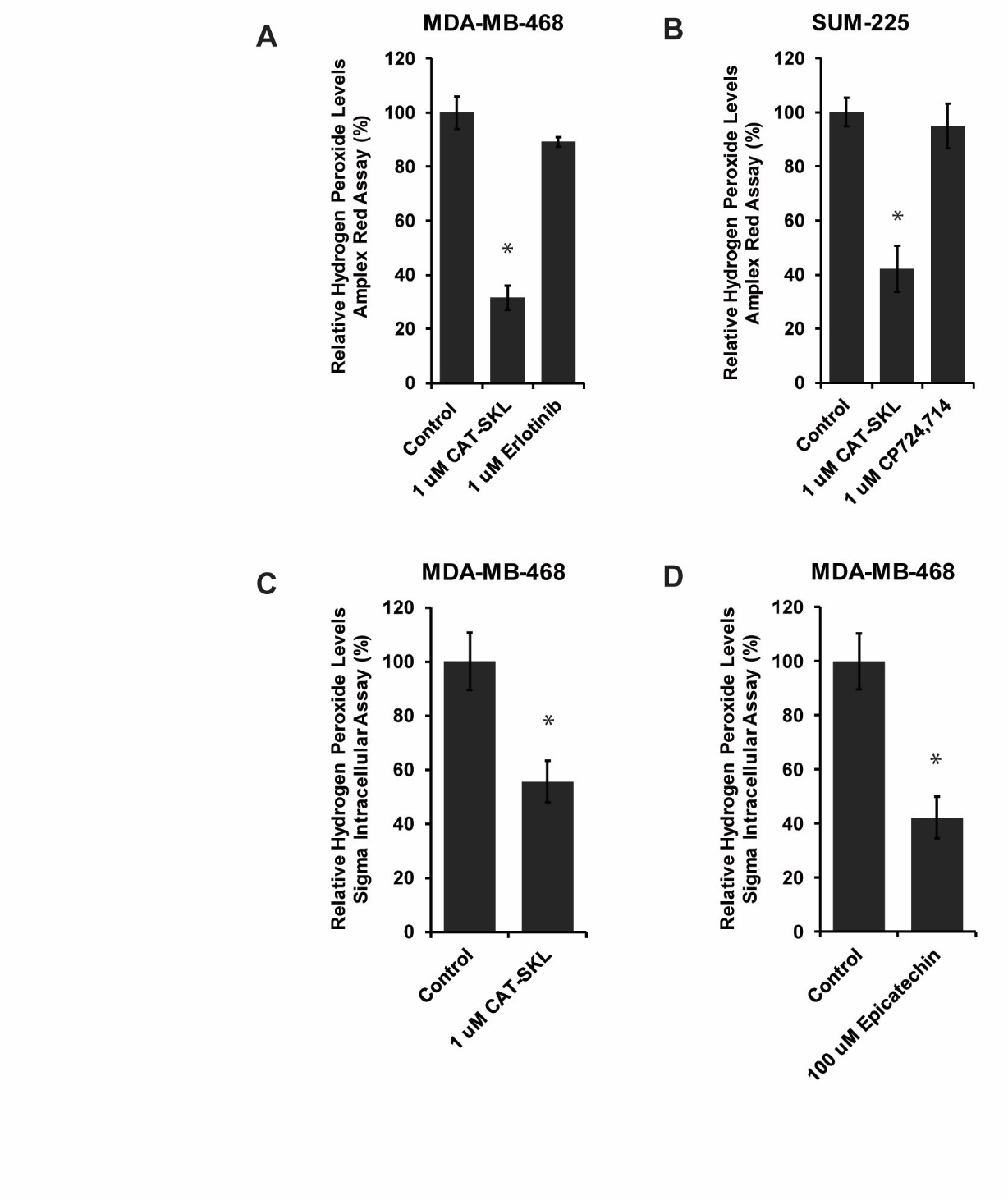


**Supplementary Figure S6.** **Effects of the various drugs or compounds used in this study on cellular ROS levels in TNBC cell cultures.** **(A)** MDA-MB-468 TNBC cell line cultures were treated with vehicle control, CAT-SKL or erlotinib for 20 hours at the indicated dosage, and ROS was measured using the Amplex Red Hydrogen Peroxide assay kit (Invitrogen). **(B)** SUM-225 cell line cultures were treated with vehicle control, CAT-SKL or CP724,714 for 20 hours, and ROS was measured using the Amplex Red Hydrogen Peroxide assay kit (Invitrogen). **(C)** MDA-MB-468 TNBC cell line cultures were treated with vehicle control or CAT-SKL for 20 hours, and ROS was measured using the Intracellular Hydrogen Peroxide assay kit (Sigma-Aldrich) approach. **(D)** MDA-MB-468 TNBC cell line cultures were treated with vehicle control or (-)-epicatechin for 20 hours, and ROS was measured using the Intracellular Hydrogen Peroxide assay kit (Sigma-Aldrich) approach. Assays were run in triplicate, and each panel is representative of 3 independent experiments.


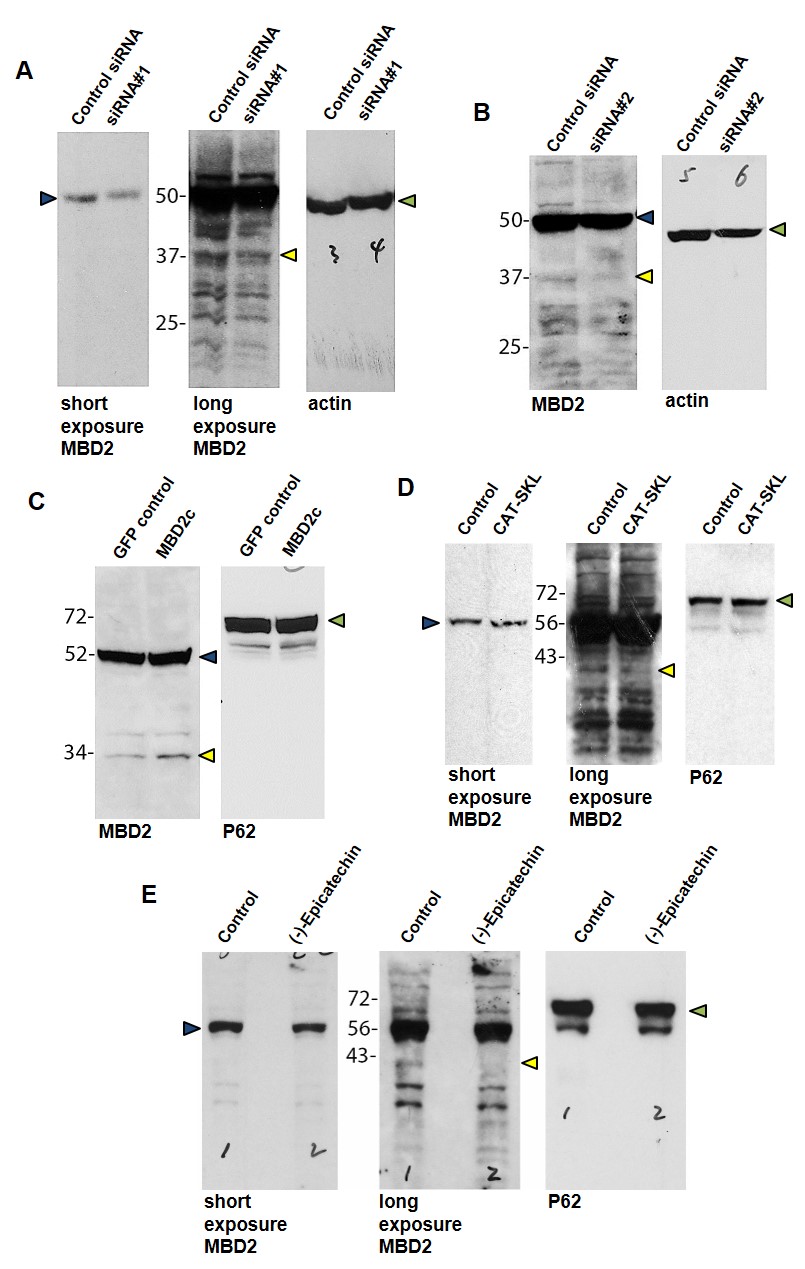


**Supplementary Figure S7. Full-length scanned images of immunoblot film.** Originals for cropped bands in: (**A**) Figure 4 panel D. (**B**) Supplementary Figure S5. (**C**) Figure 4 panel F. (**D**) Figure 5. (**E**) Figure 6. Protein ladders were used to estimate molecular weight (kilodaltons). Blue arrow indicates MBD2a band; yellow arrow indicates the MBD2c band; green arrow indicates the control gene band.

The antibody-targeted protein is indicated underneath each panel.
